# Inhibition of Nociception in a Preclinical Episodic Migraine Model by Dietary Supplementation of Grape Seed Extract Involves Activation of Endocannabinoid Receptors

**DOI:** 10.3389/fpain.2022.809352

**Published:** 2022-01-27

**Authors:** Sara E. Woodman, Sophia R. Antonopoulos, Paul L. Durham

**Affiliations:** Department of Biology, Missouri State University, Jordan Valley Innovation Center-Center for Biomedical and Life Sciences, Springfield, MO, United States

**Keywords:** cannabinoid receptor, central sensitization, CGRP, migraine, trigeminal, stress

## Abstract

Migraine is associated with peripheral and central sensitization of the trigeminal system and dysfunction of descending pain modulation pathways. Recently, dietary inclusion of grape seed extract (GSE) was shown to inhibit mechanical nociception in a preclinical model of chronic temporomandibular joint disorder, a condition often comorbid with migraine, with the antinociceptive effect mediated, in part, by activation of 5-HT3/7 and GABAB receptors. This study further investigated the mechanisms by which GSE inhibits mechanical nociception in a preclinical model of episodic migraine. Hyperalgesic priming of female and male Sprague Dawley rats was induced by three consecutive daily two-hour episodes of restraint stress. Seven days after the final restraint stress, rats were exposed to pungent odors from an oil extract that contains the compound umbellulone, which stimulates CGRP release and induces migraine-like pain. Some animals received dietary supplementation of GSE in their drinking water beginning one week prior to restraint stress. Changes in mechanical sensitivity in the orofacial region and hindpaw were determined using von Frey filaments. To investigate the role of the endocannabinoid receptors in the effect of GSE, some animals were injected intracisternally with the CB1 antagonist AM 251 or the CB2 antagonist AM 630 prior to odor inhalation. Changes in CGRP expression in the spinal trigeminal nucleus (STN) in response to stress, odor and GSE supplementation were studied using immunohistochemistry. Exposure of stress-primed animals to the odor caused a significant increase in the average number of withdrawal responses to mechanical stimulation in both the orofacial region and hindpaw, and the effect was significantly suppressed by daily supplementation with GSE. The anti-nociceptive effect of GSE was inhibited by intracisternal administration of antagonists of CB1 and CB2 receptors. GSE supplementation inhibited odor-mediated stimulation of CGRP expression in the STN in sensitized animals. These results demonstrate that GSE supplementation inhibits trigeminal pain signaling in an injury-free model of migraine-like pain via activation of endocannabinoid receptors and repression of CGRP expression centrally. Hence, we propose that GSE may be beneficial as a complementary migraine therapeutic.

## Introduction

Migraine is a prevalent painful neurological disease that is a leading cause of disability worldwide (1, 2). The commonly reported headache pain associated with migraine involves sensitization and activation of trigeminal ganglion nerves that provide sensory innervation of the head and face and transmit pain signals to the spinal trigeminal nucleus (STN) (3). Migraineurs are genetically predisposed to the development of a hyperresponsive nervous system, which is susceptible to multiple risk factors that promote peripheral and central sensitization or can initiate a migraine attack (4, 5). The most often reported migraine risk factor is stress, which increases allostatic load and if unmanaged can cause dysregulation of the ascending and descending pain modulation pathways and greatly influence disease onset, progression, and transition from an episodic to chronic state (6–8). Other reported migraine risk factors that also increase allostatic load and can initiate an attack in sensitized migraineurs include physical stimuli such as flickering lights, loud or irregular sounds, or strong, pungent odors (9–11). The descending inhibitory pain modulation pathway involves activation of serotonergic, GABAergic, and endocannabinoid receptors to modulate the excitability state of neurons and glial cells within the STN (12). In a recent study, dietary inclusion of GSE was shown to inhibit trigeminal pain signaling via involvement of 5-HT3, 5-HT7, and GABAB receptors in animals with a sensitized trigeminal system mediated by neck muscle inflammation (13). The goal of this study was to test the hypothesis that activation of endocannabinoid receptors and cellular changes in the STN are also involved to mediate the inhibitory effects of GSE in a preclinical episodic migraine model involving restraint stress and exposure to a pungent compound (14).

Episodic and chronic migraine pathology are associated with anxiety and stress, and the development of central sensitization, which lowers the threshold for activation of second order neurons in response to stimulatory molecules released from trigeminal ganglion neurons in the STN (15–17). The excitatory neurotransmitter glutamate and the neuropeptide calcitonin gene-related peptide (CGRP) are constitutively secreted from primary trigeminal ganglion neurons, and hence modulate the excitability state of STN neurons and glial cells (18). Elevated levels of CGRP in the STN are implicated in migraine pathology and the development of central sensitization (19) and blocking the neuromodulatory effects of CGRP is the basis of several anti-migraine therapies including triptans, onabotulinumtoxinA, gepants, and monoclonal antibodies (20).

Central sensitization can involve enhanced ascending pain signaling and/or a decrease in descending pain modulation (21). The descending inhibitory pathway involves activation of 5-HT3 and 5-HT7 receptors on inhibitory interneurons that stimulates release of the inhibitory neurotransmitters glycine and GABA (12, 22). The GABA receptors GABAA and GABAB are expressed on neurons and glial cells in the STN and their activation couples to a chloride channel or an inhibitory G-protein, respectively. In addition, the endocannabinoid system is involved in descending pain modulation and is known to play an important role in modulating stress and anxiety in humans (23–26) via activation of cannabinoid receptors (27, 28). The diverse physiological and cellular effects of the endocannabinoid system are mediated by the G-protein-coupled receptors CB1 and CB2, which are expressed on axon terminals and glial cells in both the central and peripheral nervous systems (29). Under normal physiological conditions, CB receptors are activated by endogenous ligands including 2-arachidonoylglycerol (2-AG) and anandamide (AEA), but also by Δ9-tetrahydrocannabinol (THC), cannabidiol (CBD), and phytocannabinoids (pCB) present in medical marijuana and different medical cannabis formulations (30). Dysfunction of this pathway is associated with migraine pathology while activation of endocannabinoid receptors is reported to be beneficial in migraineurs (31). In this study, the role of CB receptors in mediating the anti-nociceptive effect of GSE was investigated in an injury-free migraine model.

## Methods

### Animals

Adolescent Sprague Dawley male and female rats (d45; 150–225 g) were purchased from Missouri State University's Central Management Breeding Colony (Springfield, MO). Animals were housed individually in plastic rat cages with unrestricted access to both food and water in a room with 12 h/light dark cycles. All protocols were approved by Missouri State University's Institutional Animal Care and Use Committee and conducted in compliance with the Animal Welfare Act and National Institutes of Health guidelines. Concerted efforts were made to minimize suffering, as well as the number of animals.

### Preclinical Episodic Migraine Model

**Figure 1:**
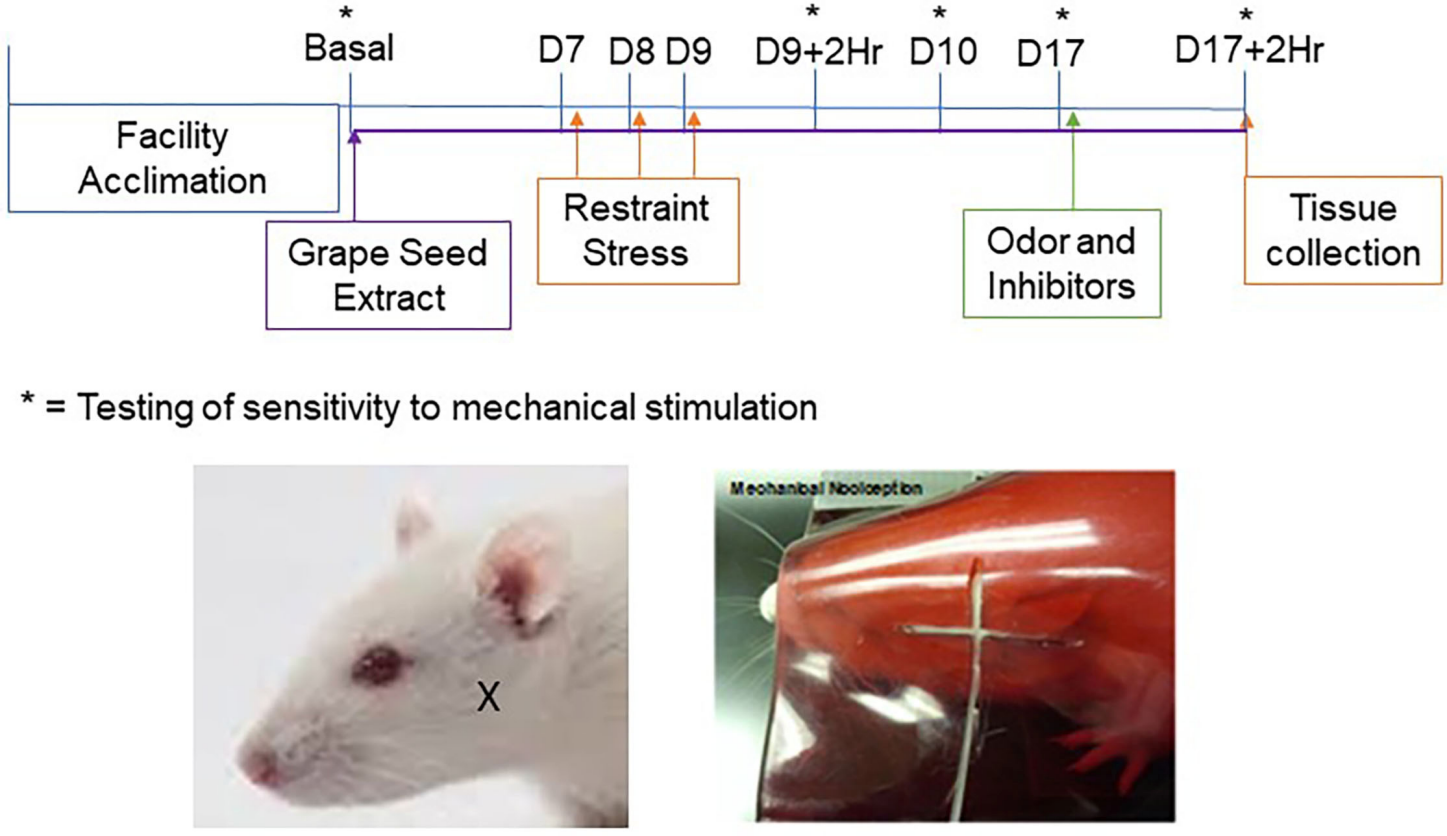
Timeline and experimental design. Dietary supplementation of GSE was initiated one week prior to restraint stress and was continued for the duration of the study. Restraint stress was performed on 3 consecutive days (D7–9) prior to injection of cannabinoid receptor inhibitors and exposure to pungent odor on day 17. Behavioral von Frey assessments were conducted on day 0 (Basal), on day 9 two hours (D9 + 2Hr) and 24 h (D10) after restraint stress, on day 17 prior to injection of inhibitors and/or odor exposure (D17), and at 2 h post exposure to the pungent odor. Tissues used for immunohistochemistry were collected after the final behavioral assessment. The location of von Frey testing in the cutaneous region over the masseter muscle is shown in the images of a rat outside the holding device (**left panel**; X marking) and a rat within the device (**right panel**; filament shown).

Our experimental design was based on the procedure used by Kopruszinski et al. (14) to promote latent sensitization of trigeminal ganglion neurons via restraint stress prior to exposure to the pungent compound umbellulone. Prior to the start of the experiment, animals were randomly sorted into three groups: naïve control animals; animals subjected to restraint stress and pungent odor; and animals that received dietary supplementation with GSE prior to restraint stress and pungent odor (Figure 1). Briefly, to induce latent sensitization of trigeminal nociceptive neurons, some animals were placed into an opaque plastic restraint tube to greatly restrict their movement for 2 h on three consecutive days (days 7–9). The length and width of the tube was chosen based on the size of the animal, to restrict movement without causing difficulty breathing, and the tube had caps on both ends that had holes to allow air to enter but prevented the animal from exiting. Animals were observed throughout the stress exposure and following stress exposure animals were returned to their home cages. After seven days, stressed animals were exposed for 10 min to the pungent volatile compounds from an oil extract of leaves from the California bay laurel tree (CBL, World Spice, Seattle, WA) that was prepared and utilized as described previously (32–34).

### Nocifensive Behavior Testing

Changes in nocifensive response to mechanical stimulation of trigeminal neurons were determined essentially as described (32–34). Prior to nociception testing, animals were allowed to acclimate to the Durham Animal Holder (UGO Basile, Gemonio, Italy) for 5 min on three consecutive days. To minimize reflexive or startle responses, animals were conditioned to a mechanical stimulus by gently rubbing the hair in the facial region above the masseter with a von Frey filament (Figure 1). Nocifensive testing over the masseter was chosen since migraine pain can present as facial pain (35, 36). The method used in our laboratory measures deep musculoskeletal pain responses rather than cutaneous, superficial, reflexive defensive responses and hence higher weight filaments are required to test for changes in nociception. Once the animals had completed 3 acclimation sessions, they were allowed to rest for 48 h prior to baseline assessments.

Mechanical nocifensive thresholds in the orofacial region over the masseter muscle were determined in male and female animals in response to a series of calibrated von Frey filaments (Stoelting, Wood Dale, IL). Two scientists blinded to the experimental conditions verified nocifensive withdrawal reactions, which were defined as a head withdrawal observed prior to the bending of the filament. Each filament was applied five times over both the right and left orofacial region of each animal and reported as an average number of reactions. Testing was done with the 100 g filament, which was chosen because it provokes a low rate of response in naïve animals but a high rate of response in sensitized animals, and the 180 g filament was used as a positive control since it consistently provokes a response even in naïve animals. Baseline measurements were established prior to experimental manipulations and additional measurements were taken 2 h, 1 day, and 7 days post restraint stress and 2 h post odor exposure. The nocifensive response to mechanical stimulation with von Frey filaments was also tested in the intraplantar region of the hindpaw of a subset of both male and female animals, with animals restrained in a plastic Durham holder set on a wire mesh platform, to allow access to their hind feet. Nocifensive withdrawal reactions were defined as foot-lifting observed prior to the bending of the filament. Lifting of the foot prior to application of the filament was not considered a positive response. Results are presented as the average number of hindpaw withdrawal responses out of 5 applications to each foot.

### Dietary Supplementation of GSE

Following basal nociceptive testing, animals designated to receive daily supplementation with GSE had their normal drinking water replaced with a 0.5% solid solution of MegaNatural®-BP GSE (Healthy Origins, Pittsburgh, PA) dissolved in water (13, 37). The animals consumed on average 800 mL of 0.5% GSE/kg/week which is equivalent to 114.3 mL/kg/day. This corresponds to 571.4 mg GSE/kg/day which is 317 ng GAE/kg/day (polyphenolic content). Animals received GSE starting one week prior to restraint stress. Dietary supplementation was continued for the duration of the study, which concluded 2 h post exposure to the pungent odor of the bay leaves. All other groups were provided with normal tap water throughout the study.

### Inhibitor Injections

Initially, some animals were lightly anesthetized using 3–5% isoflurane prior to intracisternal injection of antagonists to the CB1 and the CB2 receptors. AM251 and AM630 (Ki nM range; Tocris Bioscience, Minneapolis, MN) were dissolved in DMSO, then diluted to 500 nM in sterile 0.9% saline. Inhibitors were administered via injection of 20 μl between the occipital bone and C1 vertebrae. Animals were allowed to regain consciousness and then were exposed to the pungent odor. Following injections and odor exposure, animals were allowed to recover in their cages while being monitored for normal behaviors prior to nociception testing. As a control, some naïve and migraine model animals received an intracisternal injection of 0.9% sterile saline, 500 nM AM251, or 500 nM AM630.

### Immunohistochemistry

Upper spinal cord tissue containing the STN were removed from male and female naïve animals and from migraine model animals 2 h post exposure to the pungent odor. Briefly, animals were euthanized via CO_2_ asphyxiation and decapitation, and tissues obtained through cranial dissection. Once extracted, tissues were placed in 4% paraformaldehyde overnight at 4°C. Samples were cryoprotected by placing tissues in a 15% sucrose solution for 1 h at 4°C, and a 30% sucrose solution overnight at 4°C. Samples were then mounted in Tissue-Tek ® Optimal Cutting Temperature mounting media (Sakura® Finetek, Torrance CA), and 14 μm cross sections of the upper spinal cord were obtained using a Microm HM 525 Cryostat (Richard-Allen Scientific, Kalamazoo, MI). Sections were placed on Fisherbrand Superfrost ® Plus Microscope slides (Thermo Fischer Scientific, Waltham, MA) and stored at −20°C.

Immunostaining procedures and analysis were performed essentially as described in prior studies (38, 39). Slides containing one tissue section from each experimental condition were covered with 1X Phosphate-Buffered Saline (PBS) for 5 min prior to incubation for 20 min in PBS containing 5% normal donkey serum (Jackson ImmunoResearch Laboratories, West Grove, PA) and 0.1% Triton. A goat polyclonal antibody against CGRP (ab36001, Abcam, Cambridge, UK) was diluted 1:1000 in 5% donkey serum in PBS and incubated for 3 h at room temperature. Alexa Fluor 647 donkey anti-goat secondary antibody (Jackson Laboratories; 1:200 dilution in 5% donkey serum diluted in PBS) was then incubated with tissues for 1 h at room temperature. Tissues were mounted in Vectashield medium containing 4',6-diamidino-2-phenylindole (DAPI; Vector Laboratories, Burlingame, CA). A Zeiss Axiocam mRm camera (Carl Zeiss, Thornwood, NY) mounted on a Zeiss Imager Z1 fluorescent microscope was used to collect a single 3 x 3 tiled 200X image of the dorsal medullary horn region. Zen 2 software (Carl Zeiss) was utilized to evenly balance the background of each image. Another scientist who was blinded to the experimental conditions then conducted densitometric analysis of gray scale jpeg images using ImageJ software. For spinal cord tissues, integrated densities were acquired by measuring pixel densities in 10 non-overlapping, rectangular regions of interest (ROI) encompassing laminas I-III for each spinal cord image. Additionally, background measurements, which were acquired from acellular areas as determined by DAPI staining, were averaged and subtracted from the ROI values. Relative average means of fluorescent intensities were determined for each condition and data reported as average fold change ± SEM relative to the average mean for naive animals, which was set equal to one.

### Statistical Analysis

All data were initially evaluated for normality using a Shapiro-Wilk test. Behavioral data were found to be non-normal (*P* < 0.05), so non-parametric statistical tests were applied. To determine if nociception was different across all groups, a Kruskal Wallis test was performed. Upon reaching a significant result, a Mann-Whitney U test with a Wilcoxon's W *post-hoc* test was performed to determine if there were pairwise differences in nociception between groups at each evaluated time point. Immunohistochemical data were normally distributed, and differences between naïve tissues and tissues from animals receiving GSE were compared using an Independent Samples *T*-Test. Statistical analysis was performed using SPSS Statistical Software 25 (IBM), and changes were considered significant if *P* < 0.05.

## Results

### Effects of Restraint Stress, Pungent Odor, and GSE Administration on Nocifensive Responses

**Figure 2:**
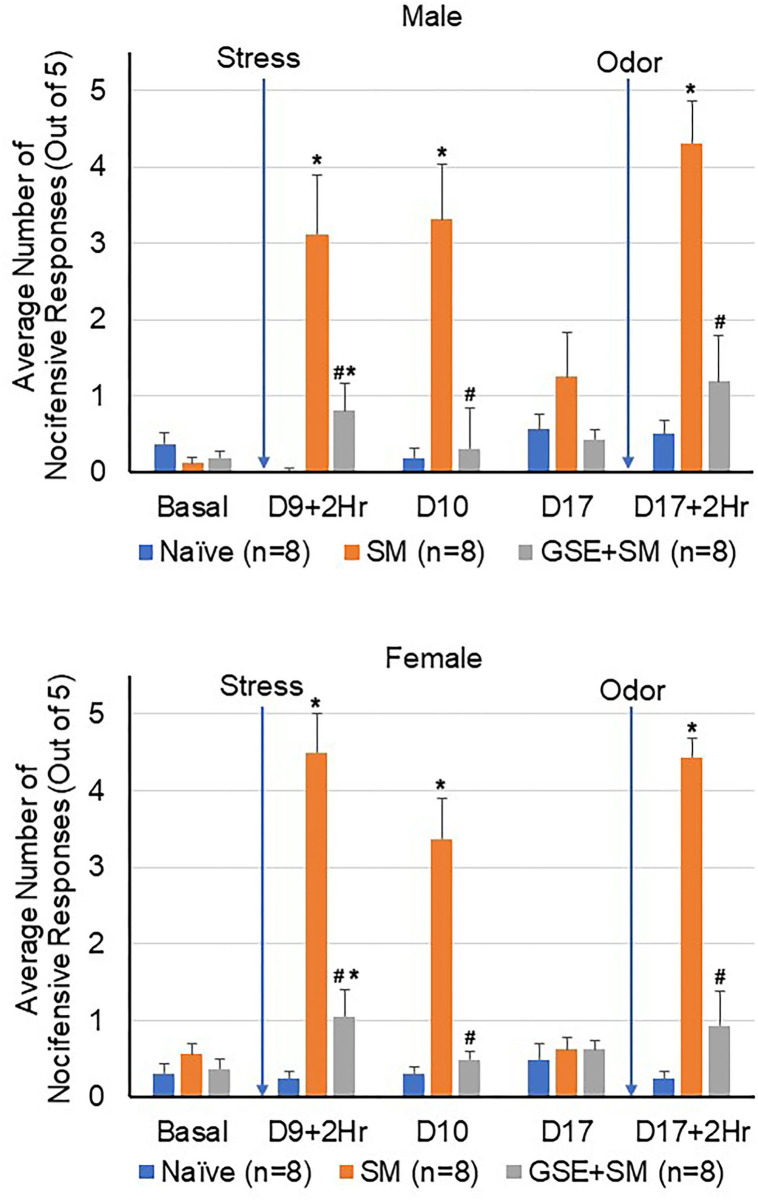
GSE supplementation inhibits trigeminal nociception in the orofacial region mediated by pungent odor in latent-sensitized animals. The average number of nocifensive head withdrawal responses ± SEM to mechanical stimuli are reported for male **(top panel)** and female animals **(bottom panel)**. Some animals were left untreated (Naïve) while other animals were subjected to restraint stress for 3 consecutive days prior to exposure to pungent odor on day 9 (stress model, SM). Some animals received daily GSE in lieu of water for 1 week prior to restraint stress and supplementation was continued for remainder of study (GSE + SM). *P < 0.05 when compared to Naïve mechanical sensitivity levels at that time point. ^#^*P* < 0.05 compared to SM levels.

Initially, the level of trigeminal nociception in response to mechanical stimulation was determined in the orofacial region with von Frey filaments in male and female animals (Figure 2). The average number of nocifensive head withdrawals in response to mechanical stimulation was <1 out of 5 applications at the basal time point for all experimental conditions in both males and females. On day 9, the nociceptive response for naïve animals remained near basal levels. However, in latent sensitized animals mediated by restraint stress, the average number of nocifensive responses was significantly (both sexes *P* = 0.001) elevated over naïve levels at 2 h and remained significantly elevated at 24 h post exposure in both male and female animals (males *P* = 0.003; females *P* = 0.001, respectively). In male and female animals receiving daily supplementation of GSE one week prior to restraint stress, the average number of nocifensive responses was significantly inhibited in sensitized animals to near basal or naïve levels (males *P* = 0.006; females *P* = 0.002). Seven days later, the average number of nocifensive withdrawals had returned to basal levels for all conditions. In latent sensitized animals mediated by restraint stress, the average number of nocifensive responses was significantly elevated over naïve levels 2 h after exposure to the pungent odor from a California bay leaf extract in both male and female animals (both sexes *P* = 0.001). However, in male and female animals receiving GSE, the average number of nocifensive responses was significantly inhibited in sensitized animals 2 h post odor exposure (males *P* = 0.015; females *P* = 0.002).

### Effects of CB1 and CB2 Antagonists on Orofacial Mechanical Sensitivity

**Figure 3:**
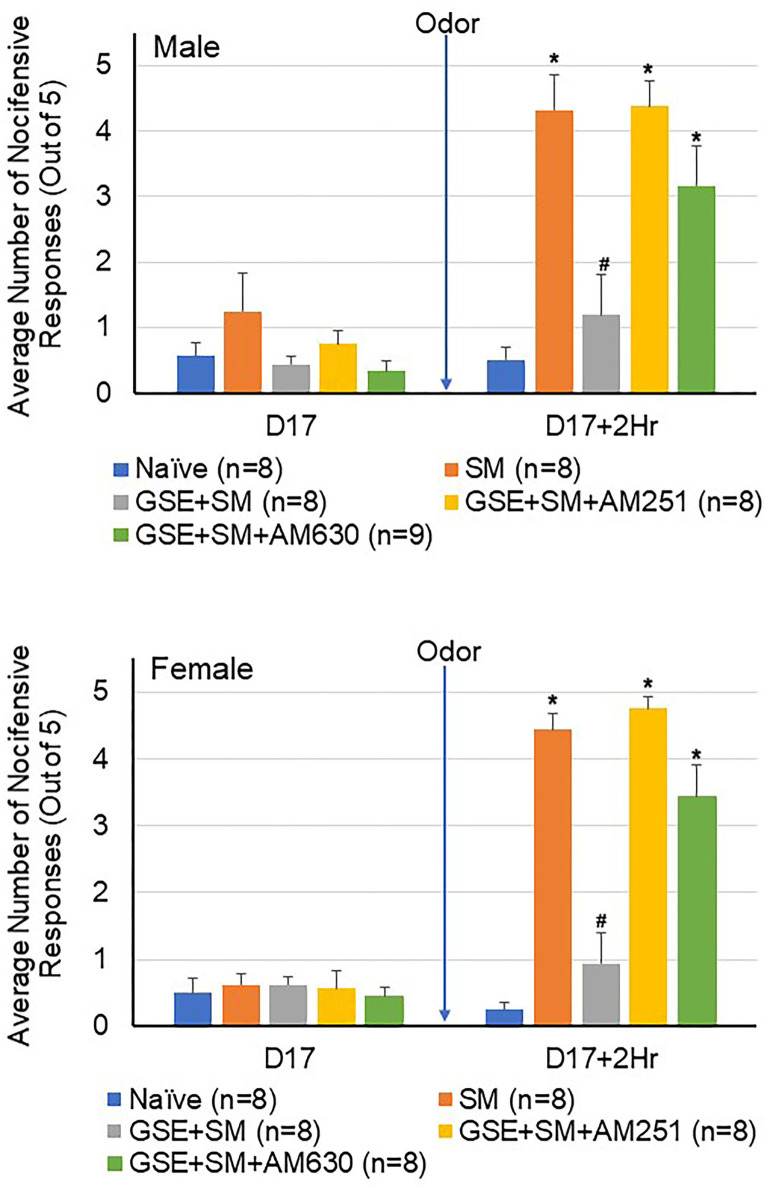
The anti-nociceptive effect of GSE involves CB1 and CB2 receptors. The average number of nocifensive head withdrawal responses to mechanical stimulation is reported for male **(top panel)** and female **(bottom panel)** animals. Some animals were left untreated (Naïve) while other animals were subjected to restraint stress (SM). Some animals received daily GSE in lieu of water for 1 week prior to restraint stress and supplementation was continued for remainder of study (GSE + SM). The GSE + SM + AM251 animals received intracisternal injection of the CB1 antagonist AM251 while the GSE + SM + AM630 animals were injected with the CB2 antagonist immediately before exposure to the pungent odor. *P < 0.05 when compared to Naive levels for that condition, ^#^*P* < 0.05 compared to SM levels.

To investigate if the anti-nociceptive effect of GSE involved activation of CB1 and CB2 receptors at the level of the STN, the CB1 antagonist AM251 or the CB2 antagonist AM630 was injected into the intracisternal space prior to stimulation with the pungent odor. As seen in Figure 3, all male and female animals exhibited similar levels of mechanical sensitivity prior to odor exposure, with average values close to naïve levels. While exposure of restraint stress sensitized animals to the odor resulted in a significant increase in the average number of nocifensive withdrawals 2 h post odor (both sexes *P* = 0.001), those animals receiving dietary GSE exhibited significantly lower response levels (males *P* = 0.006; females *P* = 0.002). However, individual administration of antagonists to CB1 and CB2 prior to odor exposure suppressed the inhibitory, anti-nociceptive effect of GSE supplementation in both male and female animals (CB1 antagonist, males *P* = 0.006, females *P* = 0.001; CB2 antagonist, males *P* = 0.014, females *P* = 0.003). Naive and sensitized animals that received saline or individual administration of CB1 or CB2 antagonists as a control did not show a significant change in mechanical sensitivity (*n* = 6).

### Mechanical Sensitivity in the Hindpaw

**Figure 4:**
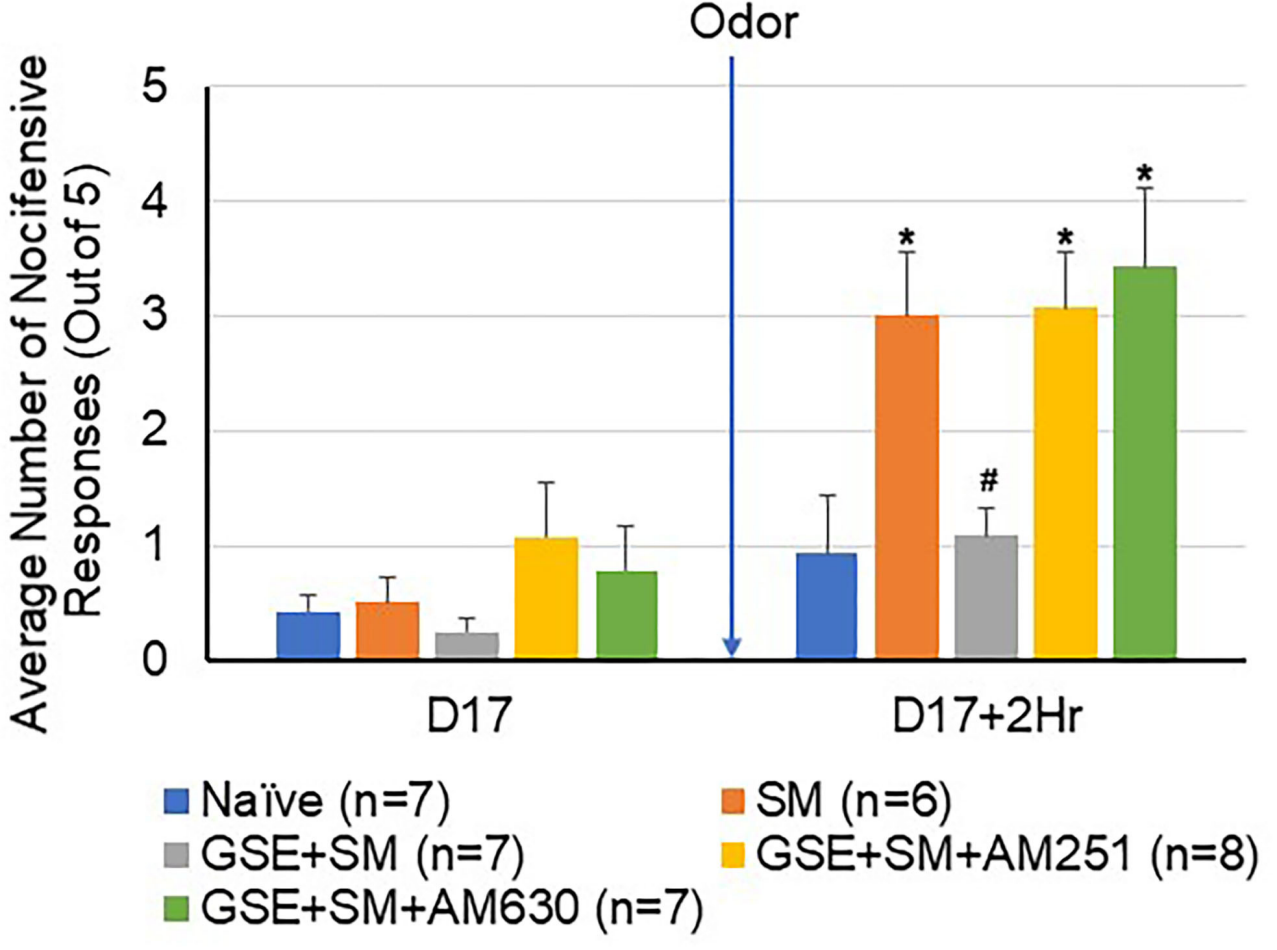
The inhibitory effect of GSE on hindpaw nocifensive behavior involves CB1 and CB2 receptors. The average number of withdrawal responses to mechanical stimulation of the hindpaw is reported for male and female animals. Some animals were left untreated (Naïve) while other animals were subjected to restraint stress (SM). Some animals received daily GSE in lieu of water for 1 week prior to restraint stress and supplementation was continued for remainder of study (GSE + SM). The GSE + SM + AM251 animals received intracisternal injection of the CB1 antagonist AM251 while the GSE + SM + AM630 animals were injected with the CB2 antagonist immediately before exposure to the pungent odor. *P < 0.05 when compared to Naive levels for that condition, ^#^*P* < 0.05 compared to SM levels.

To determine if exposure of restraint-stressed animals to the pungent odor would cause enhanced mechanical sensitivity in the hindpaw, a subset of male and female animals was tested for changes in withdrawal responses to mechanical stimulation of the hindpaw (Figure 4). Similar to the results in the orofacial region (Figure 2), a significant increase in the average number of nocifensive withdrawals was observed in the sensitized animals 2 h post odor exposure when compared to naïve levels (*P* = 0.032). Animals receiving daily GSE exhibited a significant decrease in mechanical sensitivity when compared to the stressed animals that were exposed to the odor stimulus (*P* = 0.015). Intracisternal administration of CB1 and CB2 antagonists prior to the odor stimulus was sufficient to significantly suppress the inhibitory effect of GSE and resulted in an enhanced nocifensive response to mechanical stimulation (CB1 antagonist *P* = 0.021; CB2 antagonist *P* = 0.031).

**Figure 5:**
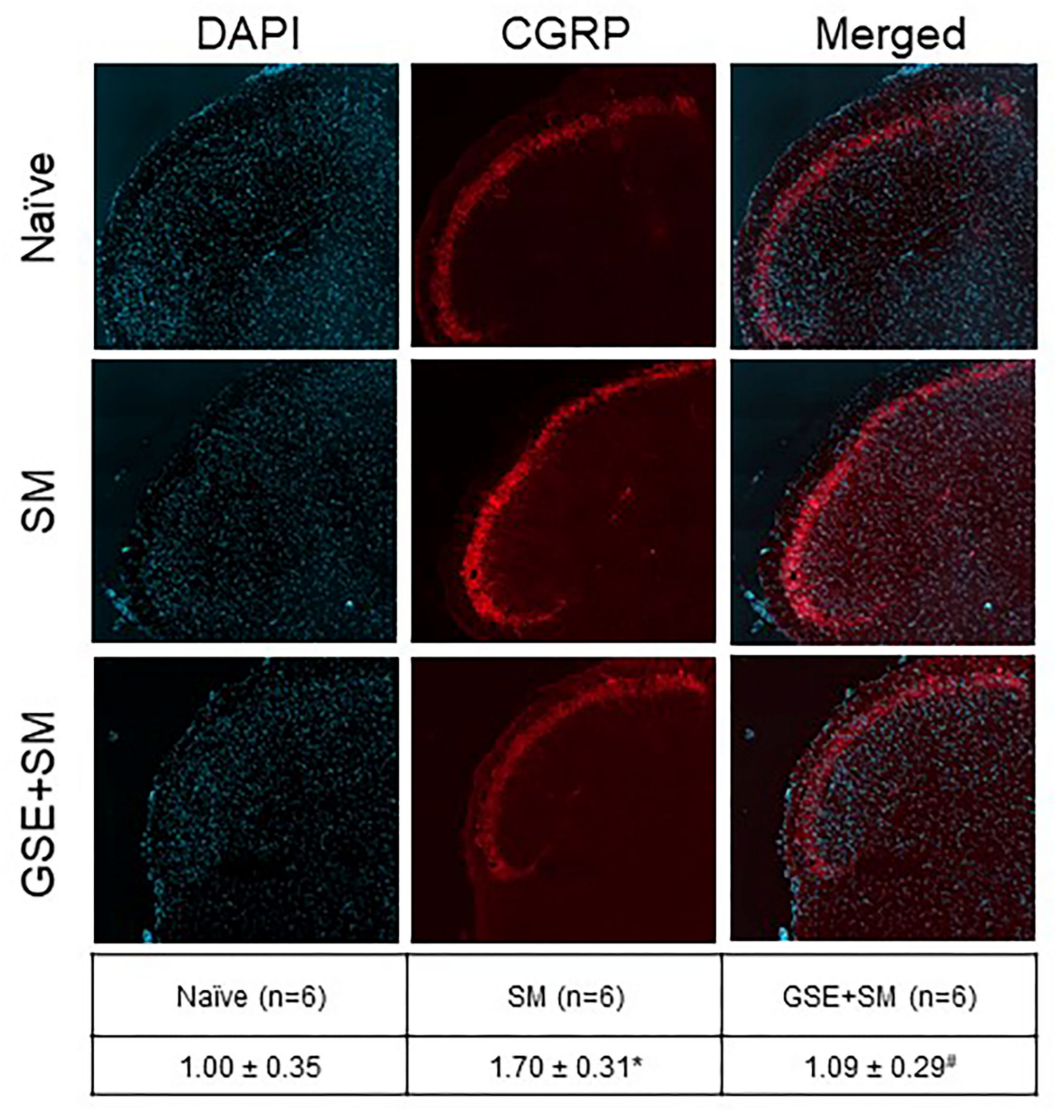
Dietary supplementation with GSE inhibits the elevated level of CGRP expression in the medullary horn mediated by exposure of restraint stressed female animals to a pungent odor. Tissues from the upper spinal cord were obtained from Naive animals, 2 h post odor exposure in stressed animals (SM), and 2 h post odor exposure in stressed animals that received GSE continuously in their drinking water (GSE + SM). Representative images of tissues costained with the fluorescent nuclear dye DAPI **(left panels)**, CGRP **(middle panels)**, and merged images **(right panels)** are shown. The average relative CGRP immunostaining intensity ± SEM as compared to levels detected in Naïve samples (mean value set equal to 1) is reported in the table. *P < 0.05 when compared to Naive levels, ^#^*P* < 0.05 compared to SM levels.

To investigate changes in CGRP expression in the STN, immunohistochemical analysis was performed on tissues obtained from female naïve animals and animals subjected to restraint stress and odor exposure and animals that received daily supplementation of GSE (Figure 5). Animals experiencing both restraint stress and inhalation of the pungent odor had significantly increased expression of CGRP (1.70 ± 0.31; *P* = 0.003) as compared to relative intensity levels in naïve control animals (1.00 ± 0.35). Animals that had restraint stress and odor exposure but had daily supplementation of GSE in their drinking water exhibited levels of CGRP expression significantly lower than non-supplemented animals (1.09 ± 0.029; *P* = 0.005), similar to basal levels observed in naïve tissues (*P* = 0.609).

## Discussion

A major finding of our study was that daily inclusion of GSE in the drinking water was sufficient to inhibit development of latent sensitization and the nocifensive response to exposure of a normally innocuous pungent odor in a preclinical model of episodic migraine. The experimental design was based on a prior study in which three consecutive days of restraint stress promoted latent sensitization of the trigeminal system in female mice such that inhalation of the compound umbellulone triggered a cutaneous allodynia response in the periorbital region and hindpaw (14). Umbellulone is a volatile organic compound present in the leaves of the California bay laurel tree, which is also referred to as the headache tree (40). This pungent compound binds and activates the transient receptor potential ankyrin 1 (TRPA1) receptor on trigeminal ganglion nociceptive neurons to stimulate CGRP release and induce nociceptive pain signaling and is implicated in migraine pathology (41–43). In the original study model, restraint stress promoted sensitization of the trigeminal system such that exposure of primed female mice to a normally subthreshold level of umbellulone facilitated pain signaling (14). In agreement, we observed a similar nocifensive response to restraint stress and exposure to the pungent odors of the bay laurel leaf in both female and male rats. Similarly, we had previously shown in a different preclinical episodic migraine model that while exposure of primed animals to the extract's volatile compounds caused enhanced nociception, inhalation in naïve, non-sensitized animals did not cause a nocifensive withdrawal response (32). In this study, we did not observe a sex difference in the rat's level of nociception in this migraine model although migraine is reported to be more prevalent in adult women than men (2). However, it should be noted that the animals used in our study were adolescent rats with an age that more closely mimics pubescent to adolescent age in humans since puberty begins 50 days after birth in rats (44). Hence, our finding may not be too surprising since in humans the prevalence of migraine is more similar in male and females during this development period (45). Significantly, dietary supplementation of GSE in the drinking water of animals for one week prior to restraint stress was sufficient to prevent latent sensitization or priming of trigeminal neurons in both female and male animals and suppress odor-induced nociception. The finding that inclusion of GSE prior to restraint stress inhibited the development of trigeminal sensitization supports the daily consumption of dietary supplements to prevent or minimize changes in the trigeminal system associated with migraine pathology.

The GSE used in this study and our prior studies (MegaNatural®-BP GSE) is a highly purified extract from *Vitis vinifera* seeds containing >90% polyphenols that exhibit anti-inflammatory properties and function as antioxidants (13, 37, 46). The inhibitory effects observed in this migraine model agree with our prior findings that dietary inclusion of GSE can suppress trigeminal sensitization and nociception in preclinical models of temporomandibular joint disorder (13), a prevalent orofacial pain condition comorbid with migraine (47, 48). In addition to GSE's ability to prevent development of a primed state, we previously reported that its inclusion as a daily supplement after establishment of prolonged trigeminal sensitization could suppress ongoing allodynia and prevent trigeminal neuron activation in a chronic orofacial pain model (13). Thus, dietary inclusion of GSE can prevent and suppress the development of trigeminal sensitization and hence functions differently than several commonly used anti-migraine therapeutics. For example, in the study by Kopruszinski et al. (14), they found that pretreatment with the centrally acting drug propranolol or the kappa opioid receptor antagonist nor-BNI prior to restraint stress could prevent both priming and transient cutaneous allodynia but not if given one hour prior to umbellulone. In that same study, the anti-migraine drug olcegepant was shown to prevent umbellulone-induced cutaneous allodynia but was not effective if given after umbellulone. In contrast, the anti-migraine drug sumatriptan inhibited the allodynic response when administered one hour post umbellulone inhalation. In a prior study, non-invasive vagus nerve stimulation (nVNS), which is recommended for the treatment of migraine, was shown to inhibit trigeminal nociception via activation of serotonergic and GABAergic receptors in the STN in a model of chronic migraine (34). Taken together, findings from our studies on GSE in preclinical orofacial pain models support the notion that bioactive molecules in the extract function to prevent or abort sensitization and activation of the trigeminal system similarly to commonly used anti-migraine drugs and nVNS. The exact mechanisms and cell types modulated by GSE are not fully elucidated but involve activation of serotonin, GABA, and endocannabinoid receptors implicated in descending pain modulation and function in a neuroprotective manner like commonly utilized anti-migraine drugs.

Migraine pathology is associated with activation of trigeminal neurons that mediate cephalic hyperalgesia and allodynia to mechanical stimuli but is also associated whole-body allodynia in other regions including extremities such as hands and feet (49–52). There is emerging evidence that migraine is a disease involving central sensitization and stress-related physiological dysregulation that plays a role in the development of allodynia in migraineurs (53). Similar to the findings of Kopruszinski et al. (14), we found exposure of restraint stressed animals to a non-noxious stimulation, pungent odor, mediated nociception in the cephalic region innervated by the trigeminal nerve but also caused an increase in mechanical sensitivity in the hindpaw. Sensory innervation of the hindpaw is provided by the dorsal root ganglion (54). Thus, the inhibitory effects of GSE are not localized to modulation of the trigeminal system but function to suppress mechanical allodynia involving DRG nociceptive neurons. This physiological response to GSE is similar to that reported for a fixed-dose combination of sumatriptan/naproxen sodium (Treximet®), which is used for the acute treatment of migraine in adults (55).

A major finding of this study was that the inhibitory effect of GSE on cephalic and hindpaw nociception in latently sensitized male and female animals could be prevented by intracisternal administration of selective antagonists of the endocannabinoid receptors CB1 and CB2. The therapeutic benefit of activation of the CB receptors in the symptomatic and prophylactic treatment of migraine is reported to involve multiple signaling pathways in the central and peripheral nervous systems to suppress inflammation and nociception (31, 56). Results from our study provide evidence that GSE contains biologically active molecules that can inhibit trigeminal nociception via activation of CB1 and CB2 receptors within the upper spinal cord. GSE's effects involving CB1 and CB2 receptors provide further support that this nutraceutical can act centrally to modulate pain signaling since we had previously shown that the inhibitory effect on trigeminal pain signaling involves activation of 5-HT3/5-HT7 and GABAB receptors in the STN (13). Furthermore, supplementation with GSE may protect against the development of a persistent pain state characteristic of chronic migraine since GSE was shown to inhibit sustained nociception in a chronic TMD model (13). Our findings provide further evidence to support an important role of CB receptors in modulating activity of the trigeminal system, which has been shown to involve suppressing nociception, repressing the stimulatory effects of CGRP, and inhibiting stimulated expression of proteins implicated in central sensitization in preclinical models of migraine (57–62). Although not a focus of this study, it is plausible that bioactive natural polyphenolic compounds present in GSE could bind to or modulate the endocannabinoid system to inhibit stress-induced sensitization of trigeminal neurons. In support of this notion, an extract from the perennial herb *Moricandia sinaica* was recently shown to exhibit analgesic and anti-inflammatory effects in rats and molecular docking studies demonstrated that polyphenols in the extract could bind CB1 and CB2 receptors (63). Further, other molecules in the GSE extract may function as full or partial agonists of CB1 and CB2 receptors as has been reported for several non-cannabinoid plant-derived natural compounds that act as cannabinoid receptor ligands (64, 65). We cannot rule out the possibility that GSE is modulating neural function in higher brain structures and possibly the periaqueductal gray and rostral ventral medulla since they play such a key role in the facilitation or inhibition of nociceptive signals and function as the final relay in the control of descending pain pathways (12). Additionally, dysregulation of the hypothalamic-pituitary-adrenal (HPA) axis and central sensitization are two physiological mechanisms that are associated with stress-induced pain disorders. The HPA axis is the primary regulator of the stress response and repeated stress can cause dysfunction of this regulatory mechanism (66). Thus, it is possible that the inhibitory effects of GSE may also involve modulation of the HPA axis.

Elevated levels of the neuropeptide CGRP are implicated in migraine pathology and modulation of CGRP release from trigeminal neurons is a primary therapeutic target of many effective anti-migraine therapies including triptans, onabotulinumtoxinA, CGRP monoclonal antibodies, and gepants (20, 67, 68). In this study, we observed a significant increase in the immunostaining intensity of CGRP in the outer lamina of the medullary dorsal horn two hours after exposure of restraint stressed animals to the pungent odor of the bay laurel leaf. The stimulatory effect of the odor is likely mediated at least in part by the compound umbellulone, which has previously been shown to cause release of CGRP and enhance nociception (40). Daily dietary supplementation of GSE significantly repressed elevated CGRP levels in the STN to basal naïve levels. This finding extends our understanding about GSE regulation of CGRP since we had previously found that supplementation with GSE could inhibit basal CGRP levels in the STN (46). Our finding is in agreement with prior studies in mice in which hyperalgesic priming of the trigeminal system in response to repetitive restraint stress was shown to be mediated by CGRP and the notion that CGRP is involved in the initiation of trigeminal neuron activation (14, 69). In addition, inclusion of GSE as a dietary supplement was shown to inhibit expression of proteins implicated in central sensitization and upregulated by CGRP in an inflammatory model of TMD (46).

In summary, the mechanisms by which GSE modulates pain pathways are likely to involve cellular events that suppress initiation and maintenance of peripheral and central sensitization, which are implicated in the pathology of migraine and other orofacial pain conditions. Our findings have provided evidence that the neuroprotective effects of GSE involve changes in the endocannabinoid system, which is activated by medical marijuana and cannabinoids. The mechanisms by which GSE functions to stimulate or inhibit cellular processes are likely mediated by the anti-inflammatory and anti-oxidant properties of the polyphenolic compounds and also modulation of receptor activity. The ability of the enriched polyphenolic GSE to prevent latent sensitization induced by repetitive stress and repress trigeminal pain signaling is in agreement with the significant cognitive benefits and neuroprotective potential of polyphenols (70). Importantly, physiologically relevant concentrations of biologically active metabolites from a water-soluble fraction of mixed berries were reported to be transported across blood brain barrier endothelial cells, and were shown to reduce neuro-inflammation and inhibit activity of pro-inflammatory biomarkers (71). Based on our findings and their results, dietary supplementation of GSE could lead to the production of bioavailable metabolites that cross the blood brain barrier and mediate changes in the STN and possibly higher brain regions involved in the stress response and trigeminal pain signaling. There are several limitations associated with our study. For example, in our study the sole source of polyphenols is from the GSE but this would not be the case in humans since coffee, tea, fruits, and other food and beverages contribute to the daily intake. Further, it is not known if humans and rats process GSE polyphenols differently and hence generate different active metabolites. However, in a recent review of the health benefits in humans, dietary inclusion of grape seed and skin extracts was reported to be neuroprotective and beneficial in other diseases (72). In conclusion, data from our studies support the notion that inclusion of GSE as dietary supplement may offer a safe and beneficial complementary therapeutic option for migraine and other orofacial pain conditions whose pathology involves persistent central sensitization and dysfunction of the descending inhibitory pain modulation pathway.

## Data Availability Statement

The raw data supporting the conclusions of this article will be made available by the authors, without undue reservation.

## Ethics Statement

The animal study was reviewed and approved by Missouri State University IACUC.

## Author Contributions

SW was primarily responsible for generation and statistical analysis of behavior data, preparation of figures, and writing and editing the manuscript. SA was primarily responsible for generation and statistical analysis of immunostaining data, preparation of figures, and writing and editing the manuscript. PD was primary responsible for the research design and final data analysis, original draft of the manuscript, and final editing of the manuscript. All authors contributed to the article and approved the submitted version.

## Funding

This work was supported by the National Institute of Health under Grant NIDCR DE024629.

## Conflict of Interest

The authors declare that the research was conducted in the absence of any commercial or financial relationships that could be construed as a potential conflict of interest.

## Publisher's Note

All claims expressed in this article are solely those of the authors and do not necessarily represent those of their affiliated organizations, or those of the publisher, the editors and the reviewers. Any product that may be evaluated in this article, or claim that may be made by its manufacturer, is not guaranteed or endorsed by the publisher.

## References

[1] Group GBDNDC. Global, regional, and national burden of neurological disorders during 1990-2015: a systematic analysis for the Global Burden of Disease Study 2015. Lancet Neurol. (2017) 16:877–97. 10.1016/S1474-4422(17)30299-5 28931491 PMC5641502

[2] Burch R Rizzoli P Loder E. The prevalence and impact of migraine and severe headache in the United States: figures and trends from government health studies. Headache. (2018) 58:496–505. 10.1111/head.13281 29527677

[3] Goadsby PJ Holland PR Martins-Oliveira M Hoffmann J Schankin C Akerman S. Pathophysiology of migraine: a disorder of sensory processing. Physiol Rev. (2017) 97:553–622. 10.1152/physrev.00034.2015 28179394 PMC5539409

[4] Manack AN Buse DC Lipton RB. Chronic migraine: epidemiology and disease burden. Curr Pain Headache Rep. (2011) 15:70–8. 10.1007/s11916-010-0157-z 21063918

[5] Su M Yu S. Chronic migraine: a process of dysmodulation and sensitization. Mol Pain. (2018) 14:1–10. 10.1177/1744806918767697 29642749 PMC5900816

[6] Borsook D Maleki N Becerra L McEwen B. Understanding migraine through the lens of maladaptive stress responses: a model disease of allostatic load. Neuron. (2012) 73:219–34. 10.1016/j.neuron.2012.01.001 22284178

[7] Maleki N Becerra L Borsook D. Migraine: maladaptive brain responses to stress. Headache. (2012) 52(Suppl. 2):102–6. 10.1111/j.1526-4610.2012.02241.x 23030541 PMC3475609

[8] Mayans L. Headache: migraine. FP Essent. (2018) 473:11–6. 30346679

[9] Friedman DI. De ver Dye T. Migraine and the environment. Headache. (2009) 49:941–52. 10.1111/j.1526-4610.2009.01443.x 19545255

[10] Hayne DP Martin PR. Relating photophobia, visual aura, and visual triggers of headache and migraine. Headache. (2019) 59:430–42. 10.1111/head.13486 30737782

[11] Silva-Neto RP Peres MF Valenca MM. Odorant substances that trigger headaches in migraine patients. Cephalalgia. (2014) 34:14–21. 10.1177/0333102413495969 23832131

[12] Ossipov MH Morimura K Porreca F. Descending pain modulation and chronification of pain. Curr Opin Support Palliat Care. (2014) 8:143–51. 10.1097/SPC.0000000000000055 24752199 PMC4301419

[13] Cornelison LE Woodman SE Durham PL. 5-HT3/7 and GABAB receptors mediate inhibition of trigeminal nociception by dietary supplementation of grape seed extract. Nutr Neurosci. (2021). 10.1080/1028415X.2021.1880211. [Epub ahead of print]. PMC8339147 33544064

[14] Kopruszinski CM Navratilova E Swiokla J Dodick DW Chessell IP Porreca F. Novel, injury-free rodent model of vulnerability for assessment of acute and preventive therapies reveals temporal contributions of CGRP-receptor activation in migraine-like pain. Cephalalgia. (2021) 41:305–17. 10.1177/0333102420959794 32985222 PMC7995998

[15] Buse DC Silberstein SD Manack AN Papapetropoulos S Lipton RB. Psychiatric comorbidities of episodic and chronic migraine. J Neurol. (2013) 260:1960–9. 10.1007/s00415-012-6725-x 23132299

[16] Grassini S Nordin S. Comorbidity in migraine with functional somatic syndromes, psychiatric disorders and inflammatory diseases: a matter of central sensitization? Behav Med. (2017) 43:91–9. 10.1080/08964289.2015.1086721 26431372

[17] Chichorro JG Porreca F Sessle B. Mechanisms of craniofacial pain. Cephalalgia. (2017) 37:613–26. 10.1177/0333102417704187 28440673

[18] Iyengar S Ossipov MH Johnson KW. The role of calcitonin gene-related peptide in peripheral and central pain mechanisms including migraine. Pain. (2017) 158:543–59. 10.1097/j.pain.0000000000000831 28301400 PMC5359791

[19] Seybold VS. The role of peptides in central sensitization. Handb Exp Pharmacol. (2009) 194:451–91. 10.1007/978-3-540-79090-7_13 19655115

[20] Reuter U. A Review of monoclonal antibody therapies and other preventative treatments in migraine. Headache. (2018) 58(Suppl. 1):48–59. 10.1111/head.13302 29697156

[21] Arendt-Nielsen L Morlion B Perrot S Dahan A Dickenson A Kress HG. Assessment and manifestation of central sensitisation across different chronic pain conditions. Eur J Pain. (2018) 22:216–41. 10.1002/ejp.1140 29105941

[22] Bannister K Dickenson AH. The plasticity of descending controls in pain: translational probing. J Physiol. (2017) 595:4159–66. 10.1113/JP274165 28387936 PMC5491855

[23] Corcoran L Roche M Finn DP. The role of the brain's endocannabinoid system in pain and its modulation by stress. Int Rev Neurobiol. (2015) 125:203–55. 10.1016/bs.irn.2015.10.003 26638768

[24] Lazary J Eszlari N Juhasz G Bagdy G. A functional variant of CB2 receptor gene interacts with childhood trauma and FAAH gene on anxious and depressive phenotypes. J Affect Disord. (2019) 257:716–22. 10.1016/j.jad.2019.07.083 31382124

[25] McKee KA Hmidan A Crocker CE Lam RW Meyer JH Crockford D. Potential therapeutic benefits of cannabinoid products in adult psychiatric disorders: a systematic review and meta-analysis of randomised controlled trials. J Psychiatr Res. (2021) 140:267–81. 10.1016/j.jpsychires.2021.05.044 34119912

[26] Petrie GN Nastase AS Aukema RJ Hill MN. Endocannabinoids, cannabinoids and the regulation of anxiety. Neuropharmacology. (2021) 195:108626. 10.1016/j.neuropharm.2021.108626 34116110

[27] Starowicz K Finn DP. Cannabinoids and pain: sites and mechanisms of action. Adv Pharmacol. (2017) 80:437–75. 10.1016/bs.apha.2017.05.003 28826543

[28] Bouchet CA Ingram SL. Cannabinoids in the descending pain modulatory circuit: role in inflammation. Pharmacol Ther. (2020) 209:107495. 10.1016/j.pharmthera.2020.107495 32004514 PMC7183429

[29] Howlett AC Abood ME. CB1 and CB2 receptor pharmacology. Adv Pharmacol. (2017) 80:169–206. 10.1016/bs.apha.2017.03.007 28826534 PMC5812699

[30] Piomelli D Beltramo M Giuffrida A Stella N. Endogenous cannabinoid signaling. Neurobiol Dis. (1998) 5(6 Pt B):462–73. 10.1006/nbdi.1998.0221 9974178

[31] Leimuranta P Khiroug L Giniatullin R. Emerging role of (Endo)cannabinoids in migraine. Front Pharmacol. (2018) 9:420. 10.3389/fphar.2018.00420 29740328 PMC5928495

[32] Hawkins JL Cornelison LE Blankenship BA Durham PL. Vagus nerve stimulation inhibits trigeminal nociception in a rodent model of episodic migraine. Pain Rep. (2017) 2:e628. 10.1097/PR9.0000000000000628 29392242 PMC5741328

[33] Cornelison LE Woodman SE Durham PL. Inhibition of trigeminal nociception by non-invasive vagus nerve stimulation: investigating the role of GABAergic and serotonergic pathways in a model of episodic migraine. Front Neurol. (2020) 11:146. 10.3389/fneur.2020.00146 32194498 PMC7066071

[34] Cornelison LE Hawkins JL Woodman SE Durham PL. Noninvasive vagus nerve stimulation and morphine transiently inhibit trigeminal pain signaling in a chronic headache model. Pain Rep. (2020) 5:e881. 10.1097/PR9.0000000000000881 33364541 PMC7752694

[35] Romero-Reyes M Uyanik JM. Orofacial pain management: current perspectives. J Pain Res. (2014) 7:99–115. 10.2147/JPR.S37593 24591846 PMC3937250

[36] Ziegeler C May A. Facial presentations of migraine, TACs, and other paroxysmal facial pain syndromes. Neurology. (2019) 93:e1138–e47. 10.1212/WNL.0000000000008124 31434691

[37] Cornelison LE Chelliboina N Woodman SE Durham PL. Dietary supplementation with grape seed extract prevents development of trigeminal sensitization and inhibits pain signaling in a preclinical chronic temporomandibular disorder model. J Oral Pathol Med. (2020) 49:514–21. 10.1111/jop.13066 32531825 PMC7474515

[38] Hawkins JL Moore NJ Miley D Durham PL. Secondary traumatic stress increases expression of proteins implicated in peripheral and central sensitization of trigeminal neurons. Brain Res. (2018) 1687:162–72. 10.1016/j.brainres.2018.03.003 29522721 PMC5882570

[39] Hawkins JL Durham PL. Enriched chicken bone broth as a dietary supplement reduces nociception and sensitization associated with prolonged jaw opening. J Oral Facial Pain Headache. (2018) 32:208–215. 10.11607/ofph.1971 29509826 PMC7001769

[40] Nassini R Materazzi S Vriens J Prenen J Benemei S De Siena G. The 'headache tree' via umbellulone and TRPA1 activates the trigeminovascular system. Brain. (2012) 135(Pt 2):376–90. 10.1093/brain/awr272 22036959

[41] Nassini R Materazzi S Benemei S Geppetti P. The TRPA1 channel in inflammatory and neuropathic pain and migraine. Rev Physiol Biochem Pharmacol. (2014) 167:1–43. 10.1007/112_2014_18 24668446

[42] Souza Monteiro de. Araujo D, Nassini R, Geppetti P, De Logu F. TRPA1 as a therapeutic target for nociceptive pain. Expert Opin Ther Targets. (2020) 24:997–1008. 10.1080/14728222.2020.1815191 32838583 PMC7610834

[43] Abdel-Magid AF. Transient Receptor Potential Ankyrin 1 (TRPA1) antagonists may provide a superior treatment for pain and migraine. ACS Med Chem Lett. (2021) 12:1193–5. 10.1021/acsmedchemlett.1c00333 34413938 PMC8365616

[44] Sengupta P. The laboratory rat: relating its age with human's. Int J Prev Med. (2013) 4:624–30. 23930179 PMC3733029

[45] Allais G Chiarle G Sinigaglia S Airola G Schiapparelli P Benedetto C. Gender-related differences in migraine. Neurol Sci. (2020) 41:429–36. 10.1007/s10072-020-04643-8 32845494 PMC7704513

[46] Cady RJ Hirst JJ Durham PL. Dietary grape seed polyphenols repress neuron and glia activation in trigeminal ganglion and trigeminal nucleus caudalis. Mol Pain. (2010) 6:91. 10.1186/1744-8069-6-91 21143976 PMC3009976

[47] Chaves TC Dach F Florencio LL Carvalho GF Goncalves MC Bigal ME. Concomitant migraine and temporomandibular disorders are associated with higher heat pain hyperalgesia and cephalic cutaneous allodynia. Clin J Pain. (2016) 32:882–8. 10.1097/AJP.0000000000000369 26905569

[48] Florencio LL de Oliveira AS Carvalho GF Dach F Bigal ME. Fernandez-de-Las-Penas C, et al. Association between severity of temporomandibular disorders and the frequency of headache attacks in women with migraine: a cross-sectional study. J Manipulative Physiol Ther. (2017) 40:250–4. 10.1016/j.jmpt.2017.02.006 28390711

[49] Noseda R Burstein R. Migraine pathophysiology: anatomy of the trigeminovascular pathway and associated neurological symptoms, CSD, sensitization and modulation of pain. Pain. (2013) 154(Suppl. 1):S44–53. 10.1016/j.pain.2013.07.021 23891892 PMC3858400

[50] Misra UK Kalita J Bhoi SK. Allodynia in migraine: clinical observation and role of prophylactic therapy. Clin J Pain. (2013) 29:577–82. 10.1097/AJP.0b013e31826b130f 23328330

[51] Fernandez-de-las-Penas C Arendt-Nielsen L Cuadrado ML Pareja JA. Generalized mechanical pain sensitivity over nerve tissues in patients with strictly unilateral migraine. Clin J Pain. (2009) 25:401–6. 10.1097/AJP.0b013e31819655b3 19454873

[52] Cuadrado ML Young WB. Fernandez-de-las-Penas C, Arias JA, Pareja JA. Migrainous corpalgia: body pain and allodynia associated with migraine attacks. Cephalalgia. (2008) 28:87–91. 10.1111/j.1468-2982.2007.01485.x 18021265

[53] Polk AN Protti TA Smitherman TA. Allodynia and disability in migraine: the mediating role of stress. Headache. (2020) 60:2281–90. 10.1111/head.14012 33169381

[54] Chen C Zhang J Sun L Zhang Y Gan WB Tang P. Long-term imaging of dorsal root ganglia in awake behaving mice. Nat Commun. (2019) 10:3087. 10.1038/s41467-019-11158-0 31300648 PMC6625980

[55] Yang LP. Sumatriptan/naproxen sodium: a review of its use in adult patients with migraine. Drugs. (2013) 73:1339–55. 10.1007/s40265-013-0099-y 23912627

[56] Greco R Demartini C Zanaboni AM Piomelli D Tassorelli C. Endocannabinoid system and migraine pain: an update. Front Neurosci. (2018) 12:172. 10.3389/fnins.2018.00172 29615860 PMC5867306

[57] Akerman S Holland PR Goadsby PJ. Cannabinoid (CB1) receptor activation inhibits trigeminovascular neurons. J Pharmacol Exp Ther. (2007) 320:64–71. 10.1124/jpet.106.106971 17018694

[58] Nagy-Grocz G Tar L Bohar Z Fejes-Szabo A Laborc KF Spekker E. The modulatory effect of anandamide on nitroglycerin-induced sensitization in the trigeminal system of the rat. Cephalalgia. (2016) 36:849–61. 10.1177/0333102415613766 26512068

[59] Akerman S Kaube H Goadsby PJ. Anandamide is able to inhibit trigeminal neurons using an *in vivo* model of trigeminovascular-mediated nociception. J Pharmacol Exp Ther. (2004) 309:56–63. 10.1124/jpet.103.059808 14718591

[60] Akerman S Kaube H Goadsby PJ. Anandamide acts as a vasodilator of dural blood vessels in vivo by activating TRPV1 receptors. Br J Pharmacol. (2004) 142:1354–60. 10.1038/sj.bjp.0705896 15277315 PMC1575202

[61] Kilinc E Ankarali S Torun IE Dagistan Y. Receptor mechanisms mediating the anti-neuroinflammatory effects of endocannabinoid system modulation in a rat model of migraine. Eur J Neurosci. (2020) 1–17. 10.1111/ejn.14897 32639078

[62] Greco R Demartini C Francavilla M Zanaboni AM Tassorelli C. Dual inhibition of FAAH and MAGL counteracts migraine-like pain and behavior in an animal model of migraine. Cells. (2021) 10:2543. 10.3390/cells10102543 34685523 PMC8534238

[63] El-Mekkawy S Shahat AA Alqahtani AS Alsaid MS Abdelfattah MAO Ullah R. A Polyphenols-rich extract from *Moricandia sinaica* Boiss. exhibits analgesic, anti-inflammatory and antipyretic activities *in vivo. Molecules*. (2020) 25:5049. 10.3390/molecules25215049 33143247 PMC7663331

[64] Gertsch J Pertwee RG Di Marzo V. Phytocannabinoids beyond the Cannabis plant - do they exist? Br J Pharmacol. (2010) 160:523–9. 10.1111/j.1476-5381.2010.00745.x 20590562 PMC2931553

[65] Goncalves ECD Baldasso GM Bicca MA Paes RS Capasso R Dutra RC. Terpenoids, cannabimimetic ligands, beyond the cannabis plant. Molecules. (2020) 25:1567. 10.3390/molecules25071567 32235333 PMC7181184

[66] Eller-Smith OC Nicol AL Christianson JA. Potential mechanisms underlying centralized pain and emerging therapeutic interventions. Front Cell Neurosci. (2018) 12:35. 10.3389/fncel.2018.00035 29487504 PMC5816755

[67] Konstantinos S Vikelis M Rapoport A. Acute care and treatment of migraine. J Neuroophthalmol. (2020) 40:472–84. 10.1097/WNO.0000000000001053 32956223

[68] Loder E Rizzoli P. Pharmacologic prevention of migraine: a narrative review of the state of the Art in 2018. Headache. (2018) 58(Suppl. 3):218–29. 10.1111/head.13375 30137671

[69] Avona A Mason BN Lackovic J Wajahat N Motina M Quigley L. Repetitive stress in mice causes migraine-like behaviors and calcitonin gene-related peptide-dependent hyperalgesic priming to a migraine trigger. Pain. (2020) 161:2539–50. 10.1097/j.pain.0000000000001953 32541386 PMC7572536

[70] Caruso G Torrisi SA Mogavero MP Currenti W Castellano S Godos J. Polyphenols and neuroprotection: therapeutic implications for cognitive decline. Pharmacol Ther. (2021) 5:108013. 10.1016/j.pharmthera.2021.108013 34624428

[71] Figueira I Garcia G Pimpao RC Terrasso AP Costa I Almeida AF. Polyphenols journey through blood-brain barrier towards neuronal protection. Sci Rep. (2017) 7:11456. 10.1038/s41598-017-11512-6 28904352 PMC5597593

[72] Sochorova L Prusova B Cebova M Jurikova T Mlcek J Adamkova A. Health effects of grape seed and skin extracts and their influence on biochemical markers. Molecules. (2020) 25:5311. 10.3390/molecules25225311 33202575 PMC7696942

